# Design and Construction of Sodium Polysulfides Defense System for Room‐Temperature Na–S Battery

**DOI:** 10.1002/advs.201901557

**Published:** 2019-09-30

**Authors:** Tingting Yang, Bingshu Guo, Wenyan Du, Muhammad Kashif Aslam, Mengli Tao, Wei Zhong, Yuming Chen, Shu‐Juan Bao, Xuan Zhang, Maowen Xu

**Affiliations:** ^1^ Key Laboratory of Luminescent and Real‐Time Analytical Chemistry (Southwest University) Ministry of Education School of Materials and Energy Southwest University Chongqing 400715 P. R. China; ^2^ Department of Nuclear Science and Engineering Department of Materials Science and Engineering Massachusetts Institute of Technology Cambridge MA 02139 USA; ^3^ Department of Materials Engineering KU Leuven Leuven 3001 Belgium

**Keywords:** defense sodium polysulfides, HCS/MoS_2_, in situ Raman, room‐temperature Na–S batteries

## Abstract

Room‐temperature Na–S batteries are facing one of the most serious challenges of charge/discharge with long cycling stability due to the severe shuttle effect and volume expansion. Herein, a sodium polysulfides defense system is presented by designing and constructing the cathode‐separator double barriers. In this strategy, the hollow carbon spheres are decorated with MoS_2_ (HCS/MoS_2_) as the S carrier (S@HCS/MoS_2_). Meanwhile, the HCS/MoS_2_ composite is uniformly coated on the surface of the glass fiber as the separator. During the discharge process, the MoS_2_ can adsorb soluble polysulfides (NaPSs) intermediates and the hollow carbon spheres can improve the conductivity of S as well as act as the reservoir for electrolyte and NaPSs, inhibiting them from entering the anode to make Na deteriorate. As a result, the cathode‐separator group applied to room‐temperature Na–S battery can enable a capacity of ≈1309 mAh g^−1^ at 0.1 C and long cycling life up to 1000 cycles at 1 C. This study provides a novel and effective way to develop durable room‐temperature Na–S batteries.

## Graphical abstract

An efficient polysulfides defense system in which the cathode‐separator is constructed by HCS/MoS_2_ composite is proposed and designed. Benefiting from the structural and compositional advantages, the S@HCS/MoS_2_ composite cathode shows excellent electrochemical performance.

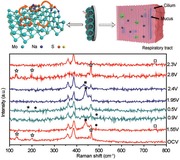

## 1 Introduction

The Rechargeable battery is an essential power technology for mobile electronic, vehicles and power grid energy storage. The popularization and application of this technology can not only reduce the environmental pollution caused by burning fossil fuels, but also address the issue of the intermittency of green energy resources including wind and solar energy, providing convenience for the life and development of human society. Therefore, it is crucial to explore and develop an energy storage system which is capable of supplementing existing limited energy density and long cycle life of lithium‐ion battery (LIBs).^1^, ^2^, ^3^ Recently, room‐temperature Na–S batteries have attracted much attention because of their high theoretical specific capacity (1675 mAh g^−1^) and energy density (1274 Wh kg^−1^) as well as abundant Na and S resources in the Earth's crust.^4^, ^5^, ^6^, ^7^ These advantages make the room‐temperature Na–S battery have broad application prospects in large‐scale power grid equipment.

However, there are still some burning questions that need to be solved in room‐temperature Na–S battery: first, the active S has poor conductivity, resulting in slow electrochemical reaction kinetics and low utilization.^8^, ^9^ Second, Na–S batteries have a higher volume expansion than lithium–sulfur batteries (LSBs), which makes the cathode structure of Na–S batteries prone to collapse.^10^ Finally, sodium polysulfides generated in the multistep reaction have high reactivity and solubility and are easy to diffuse to the sodium anode, resulting in serious “shuttle effect” that leads to a significant reduction in capacity.^11^, ^12^, ^13^

**Figure 1:**
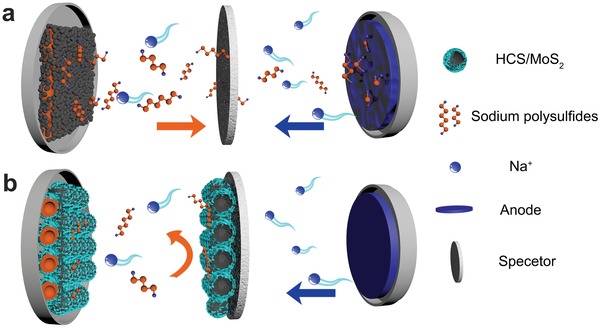
Schematic diagram of a) the S/C composite and b) the S@HCS/MoS_2_ electrode with modified glass fiber during the discharge process.

Some progresses have been made in improving the poor conductivity of S and reaction kinetics of room‐temperature Na–S batteries.^14^, ^15^, ^16^ For example, porous carbon materials with good electrical conductivity can be combined with S to enhance the electrical conductivity of the cathode, such as carbon nanofiber,^17^, ^18^ carbon cloth,^19^ carbon nanotube,^20^, ^21^ etc. Introducing carbon matrix can not only improve the utilization of S, but also are able to efficiently accommodate the volume expansion due to their abundant porous structure. For example, Wang et al. have constructed a Na–S battery using S@interconnected mesoporous carbon hollow nanospheres as the cathode. The results showed that the battery can deliver a good capacity of ≈390 and 127 mAh g^−1^ at 0.1 and 5 A g^−1^, respectively. But these batteries can only cycle for 200 cycles.^22^ Although the S/carbon hybrid design can immensely improve the utilization of S, it has to be pointed out that it is difficult to achieve complete electrochemical reversibility because the nonpolar carbon‐based materials have weak interaction with polar polysulfides, which cannot effectively limit the diffusion of NaPSs.^23^ As shown in **Figure** **1**a, for a typical S@carbon cathode, the high‐ordered polysulfides (Na_2_S*_x_*, 4 ≤ *x* ≤ 8) will move back and forth between the anode and cathode (known as the “shuttle effect”), and thus undergo unwanted redox reactions at the sodium metal surface.

In order to solve the severe shuttle effect in Na–S battery, the effective solution is to select certain polar materials as the sulfur host to reduce the shuttle of polysulfides, such as TiO_2_,^18^ VO_2_,^24^ MnO_2_,^25^ and Co_9_S_8_,^26^ etc. Recently, layered transition metal sulfur‐based materials, especially layered molybdenum disulfide (MoS_2_), have received increasing attention in various fields due to their excellent chemical and physical properties.^27^ As an analog of inorganic graphene, the MoS_2_ monolayer contains a layer of Mo atoms sandwiched between two S atoms, each Mo atom is bonded to six S atoms in a triangular prism arrangement. In addition to its layered structure, the polysulfides adsorption by MoS_2_ has also received wide attention. For example, Tang et al. have reported the preparation of MoS_2_ thin film as a protective barrier for polysulfides in alkaline‐sulfur batteries.^28^

However, using individual polar materials to reduce the solubility of NaPSs in Na–S battery electrolytes is not obvious.^29^ Typically, when a single polar material is combined with S, room‐temperature Na–S battery exhibits a very high initial capacity, while rapidly declines as the number of cycles increasing. This may be due to the poor electrical conductivity of polar materials, and some dissociative polysulfides can still enter the anode electrode through the separator during cycling. In consequence, designing and synthesizing the composite consisting of carbon and polar material is a feasible route. Finally, according to the structure of the battery, if the NaPSs generated in the process of charging and discharging can be effectively limited to the cathode‐separator side, while allowing Na ions move in the whole battery internal,^30^ this will be a good solution to improve electrochemical performance of room‐temperature Na–S battery.

According to the above statements, we design a hollow carbon sphere/molybdenum disulfide (HCS/MoS_2_) composite as the S host. First, the structure of the hollow carbon sphere enables it to be used as a nanocontainer for loading S, which can effectively improve S conductivity and alleviate the serious volume expansion. Then, by loading the polar MoS_2_ layer on the surface of the carbon spheres, the MoS_2_ can absorb NaPSs and reduce the dissolution of polysulfides in electrolytes. This hierarchical carbon/MoS_2_ structure forms the first defense against polysulfides. In addition, to further consolidate the polysulfides defense system, the last defender can be formed by coating the HCS/MoS_2_ composite on one side of the separator. In the discharge process, when the NaPSs overflow from the cathode and dissolve in the electrolyte, the HCS/MoS_2_ composite loaded on the separator can effectively adsorb the polysulfides and prevent the polysulfides from entering the anode, as shown in Figure 1b. Therefore, the unique cathode‐separator defense system will effectively prevent the shuttle of polysulfides, thus the Na–S battery exhibits remarkable electrochemical performance with a high specific capacity, excellent cycle life, and good coulombic efficiency.

## 2 Results and Discussion

The synthetic route of the S@HCS/MoS_2_ hierarchical sphere is illustrated in Figure S1 in the Supporting Information. First, SiO_2_ as the template was dispersed in the Tris‐buffer solution (10 × 10^−3^ m, pH = 8.5) and then stirred with dopamine hydrochloride (DAH) to form the SiO_2_@PDA. As‐prepared SiO_2_@PDA composite was annealed at 700 °C under H_2_. The SiO_2_ template can be removed by hydrofluoric acid to form HCS, followed by a hydrothermal reaction to grow the MoS_2_ nanosheets on the HCS surface. The HCS/MoS_2_ composite was obtained after further annealing in Ar (90%)/H_2_ (10%) at 700 °C. Finally, S was injected into the HCS/MoS_2_ matrix to form the S@HCS/MoS_2_ composite. The average diameter of the amorphous SiO_2_ template spheres is ≈130 nm (Figures S2 and S3, Supporting Information).

**Figure 2:**
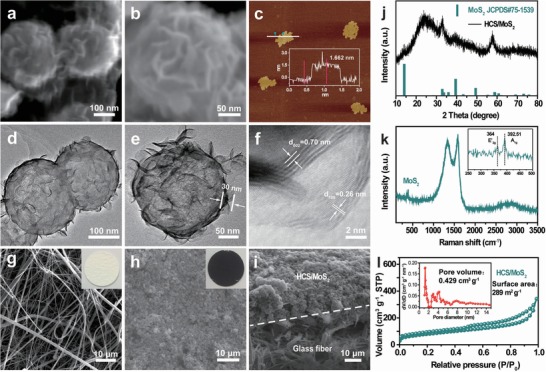
a,b) FESEM, c) AFM, d–f) TEM images of the HCS/MoS_2_, g–i) SEM images of glass fiber with and without HCS/MoS_2_ coating. j) XRD, k) Raman spectra, l) Brunauer–Emmett–Teller method (BET) adsorption and desorption curves of the HCS/MoS_2_ composite, the inset is pore‐size distribution curve.

The structure of the prepared composites was further identified by field‐emission scanning electron microscopy (FESEM) and transmission electron microscopy (TEM). Figure S4 in the Supporting information shows the morphology of HCS with a size of ≈140 nm. After the intense sonication, we can clearly find the hollow inner of HCS, indicating that the SiO_2_ template spheres have been completely removed by hydrofluoric acid. **Figure** **2**a,b shows high‐resolution FESEM images of HCS/MoS_2_. After the hydrothermal reaction, it can be clearly seen that the HCS is evenly covered with MoS_2_ nanosheets and the average size of HCS/MoS_2_ is ≈180 nm. The thickness of the MoS_2_ nanosheets was evaluated by an atomic force microscope (AFM) analysis in Figure 2c. The selected nanosheet area indicates that the thickness of the MoS_2_ nanosheet is 1.662 nm. Moreover, the morphology and microstructure of nanostructured HCS/MoS_2_ can be controlled by changing their preparation parameters (Figure S5, Supporting Information).

The hollow‐shell hierarchical structure of the HCS/MoS_2_ composite was further confirmed by TEM images. The MoS_2_ nanosheets are closely attached to the surface of HCS to form the outer shell. In Figure 2d,e, the shell thickness of HCS/MoS_2_ is ≈30 nm. High‐resolution TEM images show that each MoS_2_ nanosheet is composed of several layers. It can be observed that the lattice fringes are visible at the edge of the nanosheet, and the interface distances are 0.26 and 0.70 nm, respectively, which can be ascribed to the (100) and (002) crystal faces of the hexagonal MoS_2_ (Figure 2f).^27^ The HCS can not only reduce the volume strain of NaPSs during cycling but also can increase the conductivity of S. Meanwhile, nanocarbon subunits are able to reduce the transmission resistance of electrons and ions upon cycling, thereby maximizing the utilization of S. In addition, polar MoS_2_ nanosheets on the surface of the carbon spheres for effective adsorption of NaPSs reduce the shuttle of intermediates to prolong the life cycle of the battery.

Furthermore, the HCS/MoS_2_ composite is not only used as the S host but also prevents from the shuttle of NaPSs when coated on glass fiber separators. The corresponding SEM images of individual glass fiber and HCS/MoS_2_/glass fiber composite separator were presented in Figure 2g–i. It can be seen that the gaps between the glass fibers are very large, up to several hundred micrometers in diameter (Figure 2g), which makes the polysulfides easy to pass through during the charging and discharging of room‐temperature Na–S battery. However, the pores are completely covered after the uniform coating of HCS/MoS_2_ (Figure 2h). The cross‐section image further displays that the membrane is composed of closely stacked HCS/MoS_2_ composite with a thickness of around 10 µm (Figure 2i).

To confirm the purity and crystal phase, the composite was further characterized by X‐ray diffraction (XRD). The peaks at 14°, 33°, 40°, and 58° of the HCS/MoS_2_ pattern correspond to the (002), (100), (103), and (110) faces in the hexagonal MoS_2_ (JCPDS no. 37–1492), respectively.^31^ Meanwhile, a peak of 23.7° shows the carbon matrix of the HCS in Figure 2j. Raman spectra were also recorded to study the crystallinity of carbon in HCS (Figure S6b, Supporting Information) and HCS/MoS_2_ (Figure 2k). There are two peaks located at 1335 and 1585 cm^−1^ of HCS/MoS_2_ in the Raman spectra, corresponds to the D and G bands, respectively. The value of *I*_D_/*I*_G_ is 0.95 indicating that the HCS/MoS_2_ has good graphitization characteristics. Furthermore, the two peaks at 364 cm^−1^ (E′_2 g_) and 392 cm^−1^ (A_1g_) are characteristic peaks of MoS_2_.^32^ The HCS (Figure S6c,d, Supporting Information) exhibits high surface area of ≈644 m^2^ g^−1^ with a pore volume of 0.783 cm^3^ g^−1^. After coating MoS_2_, the surface area of HCS/MoS_2_ decreases to 289 m^2^ g^−1^ with a pore volume of 0.429 cm^3^ g^−1^ (Figure 2l).

**Figure 3:**
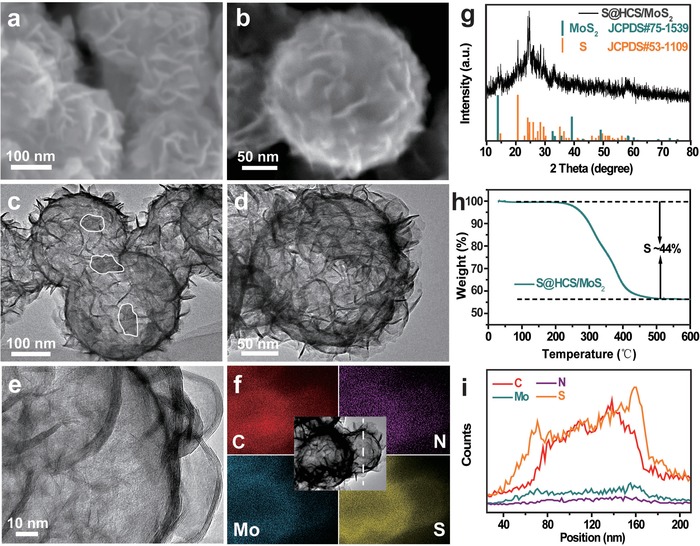
a,b) FESEM, c–e) TEM, f) EDS elemental mappings, g) XRD, h) TGA, i) linear elemental distributions of the S@HCS/MoS_2_ composite.

Sulfur was injected into the HCS/MoS_2_ composite by the method of melting and diffusion. It can be seen from **Figure** **3**a,b that the morphology of the composite remains intact. According to the TEM (Figure 3c–e), it can be clearly seen that the cavity of S@HCS/MoS_2_ shows a dark color, and the shaded portions of the partial blocks show S successfully diffused into the hollow spheres. Figure 3g is the XRD pattern of the S@HCS/MoS_2_. The comparison with the standard card of S (JCPDS no. 53–1109) indicates that S has been successfully injected into the HCS/MoS_2_. The TGA curve in Figure 3h shows that sulfur content in the compound is about 44 wt%. In Figure S7 in the Supporting Information, the BET surface area and pore size of S@HCS/MoS_2_ are 29 m^2^ g^−1^ and 0.068 cm^3^ g^−1^, respectively. By distributing S in the hollow carbon sphere uniformly, the conductivity of sulfur can be promoted and the charge transfer dynamics can be improved. It can be observed from Figure 3f,g that elements of C, N, Mo, and S can be observed in the S@HCS/MoS_2_ composite.

**Figure 4:**
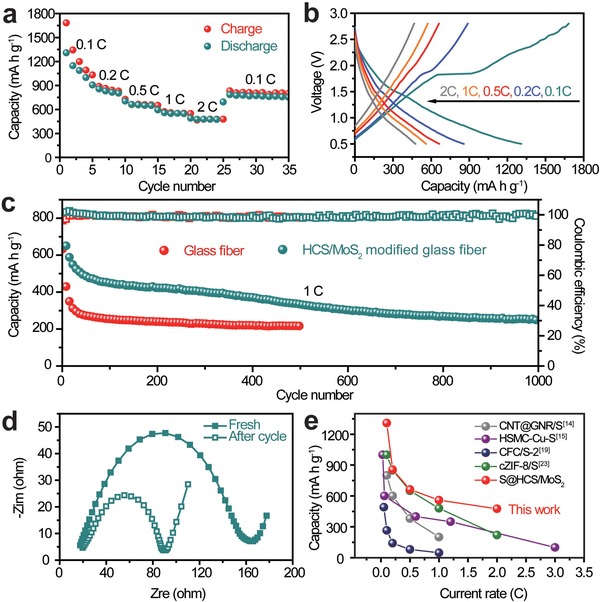
a) Rate capacities, b) voltage profiles, c) cycling performance, and d) Nyquist plots of the S@HCS/MoS_2_ composite before and after cycles. e) Rate comparation between the published literatures^14^, ^15^, ^19^, ^23^ and the S@HCS/MoS_2_ electrode developed in this work.

The Na–S battery using S@HCS/MoS_2_ composite as a cathode and HCS/MoS_2_ modified glass fiber as a separator was assembled at room temperature in the glovebox and its electrochemical performance was characterized. The rate capability of the S@HCS/MoS_2_ cathode can be seen in **Figure** **4**a. At different current densities of 0.1 C, 0.2 C, 0.5 C, 1 C, and 2 C, the discharge capacities of the cathode are 1309, 856, 663, 559, and 476 mAh g^−1^, respectively. Figure 4b shows the voltage distribution of continuous current discharge and charge at different current densities. The capacity of HCS/MoS_2_ composite under the same conditions was shown in Figure S8b in the Supporting Information.

The long‐term cycling (Figure 4c) was performed at 1 C to confirm the stability of the S@HCS/MoS_2_ cathode, with HCS/MoS_2_ coated separator and with common glass fiber. The S@HCS/MoS_2_ exhibits as high as 1090 mAh g^−1^ initial capacity and the high coulombic efficiency of about 100% can be maintained over 1000 cycles. The capacity of the long cycle first stabilized and then decreased slightly. This may be due to the deposition of NaPSs on the surface of the modified separator during long‐term charge and discharge, resulting in weakened adsorption of HCS/MoS_2_ modified glass fiber. In contrast, the S@HCS/MoS_2_ (S loading of ≈35 wt%) cathode with ordinary glass fiber (Figure 4c; Figure S9, Supporting Information) has a relatively low initial capacity of 633 mAh g^−1^, and the capacity decays faster in the next cycles with only 216.5 mAh g^−1^ in the 500th cycle. Figure S10 in the Supporting Information shows that the cell polarization is smaller after the HCS/MoS_2_ modification, and the discharge capacity increases from 633 to 1090 mAh g^−1^, showing the improved utilization of active materials. In addition, the capacity of the S@HCS (S loading of ≈40 wt%) is obviously lower than that of the S@HCS/MoS_2_ (Figure S11a, Supporting Information), and the inset shows that the glass fiber deteriorates severely after cycles. Figure S11b in the Supporting Information shows the impedance before the S@HCS battery cycle, which further indicated that the electrical conductivity of S@HCS was insufficient, and S with poor electrical conductivity did not fully react, thus showing a lower capacity. It is worth to mentioning that the performance of battery can be changed by controlling the thickness of the HCS@MoS_2_ in cathode and separator (Figure S12, Supporting Information). The impedance tests were performed before and after cycling. As shown in Figure 4d, the semicircle of the electrode after cycle (*R*_ct_ = 90 Ω) is smaller than the new static batteries (*R*_ct_ = 165 Ω). And the *R*_ct_ decreases by around 45% after cycles, indicating that the HCS/MoS_2_ composite could enhance the charge transfer ability of Na–S batteries.

The corresponding glass fiber also reflects the dissolution of polysulfides. For the battery assembled by common glass fiber, not only the polysulfides dissolved in the electrolyte appear yellow, but also the separator in contact with Na anode is significantly deteriorated under the severe shuttle effect (Figure S13a, Supporting Information). However, the glass fiber uniformly coated with HCS/MoS_2_ can be well maintained, with mildly yellow color of polysulfides solution, which indicates that the HCS/MoS_2_ composite effectively isolates the polysulfides in the cathode electrode and prevents the polysulfides from shuttling to Na anode. On the other side of the separator, the HCS/MoS_2_ coating on the surface can still be well seen (Figures S13b and S14, Supporting Information). Besides, the FESEM image in Figure S13c in the Supporting Information shows that the S@HCS/MoS_2_ hierarchical spheres are unbroken after the cycling. Correspondingly, the sodium from the cell with HCS/MoS_2_ coated separator has a relatively smoother surface (the inset in Figure S15 in the Supporting Information), and its EDS mappings spectrum further declares that the shuttle effect is hardly happened compared with S@HCS/MoS_2_ with common glass fiber. Compared to the reported results,^14^, ^15^, ^19^, ^23^ the S@HCS/MoS_2_ electrode shows excellent rate performance at a wider range of current density (0.1–2 C) in Figure 4e, which is better than other reported S cathodes (Table S1, Supporting Information).

**Figure 5:**
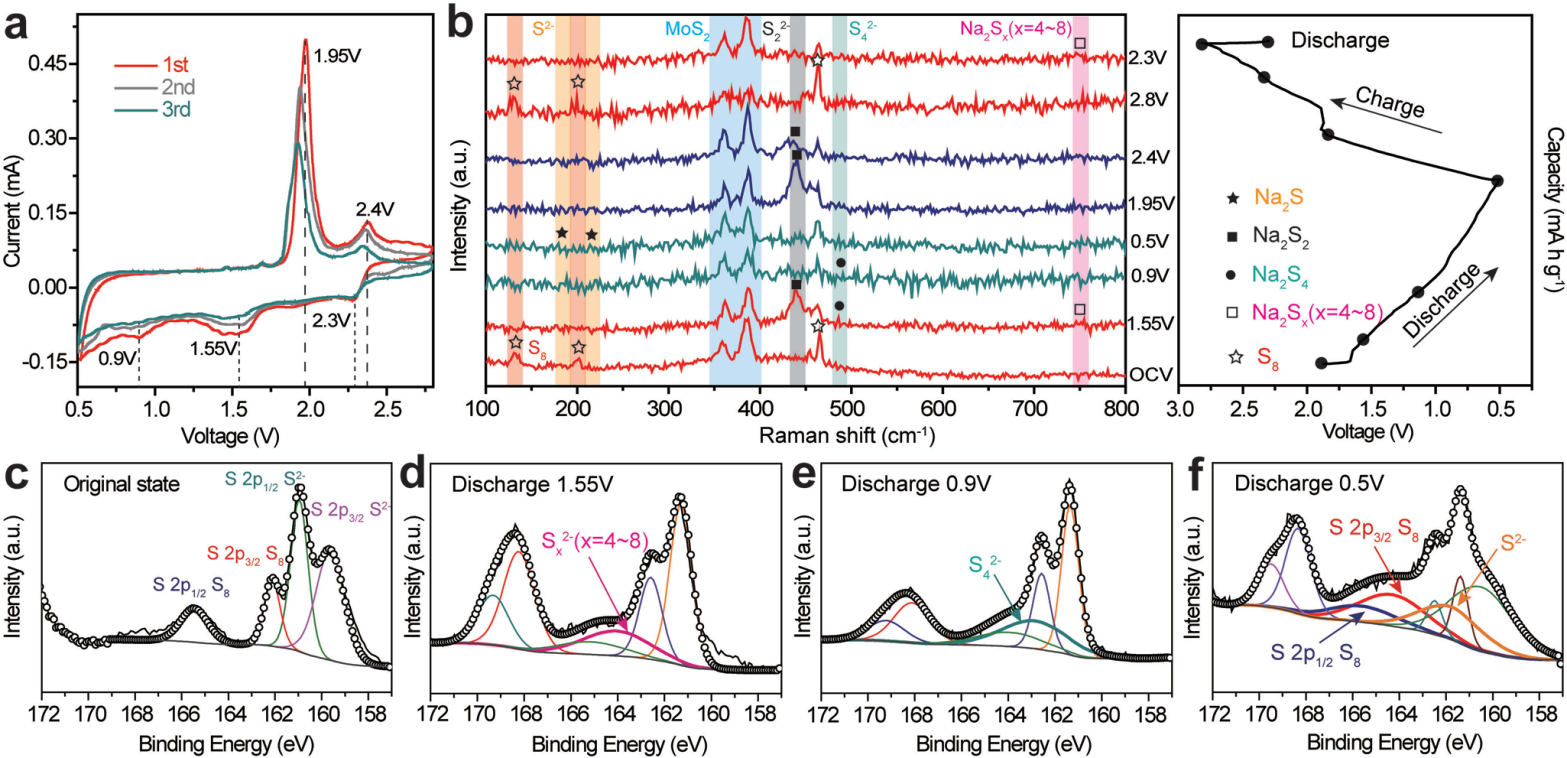
a) CV curves and b) in situ Raman analysis of the S@HCS/MoS_2_ electrode at different discharge (OCV, 1.55, 0.9, 0.5, and 2.3 V) or charge (1.95, 2.4, and 2.8 V) stages. c–f) Ex‐situ XPS analysis of the S@HCS/MoS_2_ electrode at different discharge (Original, 1.55, 0.9, and 0.5 V) stages.

**Figure** **5**a is the cyclic voltammetry (CV) curves of the battery at a sweep rate of 0.1 mV s^−1^ in the first three cycles. In the cathode scanning, the two reduction peaks at 2.3 and 1.55 V correspond to the conversion of sulfur (S_8_) to long‐chain polysulfides (Na_2_S*_x_*, *x* = 4–8) and the conversion of long‐chain sodium polysulfides to short‐chain sodium polysulfides (Na_2_S_2_/Na_2_S), respectively.^33^ The reduction peak at ≈0.9 V in CV curves of S@HCS/MoS_2_ with modified separator (Figure S16, Supporting Information) can be due to the irreversible reaction of Na^+^ in MoS_2_ intercalation.^1^, ^34^

From the in situ Raman spectrum (Figure 5b), at the open circuit voltage, in addition to the E′_2 g_ and A_1g_ characteristic peaks of MoS_2_, the peaks at 140, 200, and 465 cm^−1^ are shown as peaks of S. Figure 5c is an X‐ray photoelectron spectroscopy (XPS) spectrum of S@HCS/MoS_2_. Correspondingly, two peaks of S 2p^3/2^ and S 2p^1/2^ of divalent sulfide ions (S^2−^) appear at 159.7 and 161 eV in Figure 5c, which are in agreement with the XPS results of MoS_2_ reported in the literature.^35^ Meanwhile, S 2p^3/2^ and S 2p^1/2^ of elemental sulfur (S_8_) correspond to two double peaks at 162.1 and 165.5 eV in the spectrum of the HCS/MoS_2_ composite. When the battery is discharged from the initial voltage to 1.55 V, the peaks at 445 and 750 cm^−1^ of the Raman spectrum show the appearance of Na_2_S_2_ and Na_2_S*_x_* (*x* = 4–8), and the 164 eV peak in the XPS spectrum also corresponds to the long‐chain polysulfides (S*_x_* ^2−^) in Figure 5d.^6^ At 0.9 V, the peaks of the Raman spectra at 470 cm^−1^ correspond to Na_2_S_4_.^10^ Besides, the peak at 163 eV in Figure 5e also shows the presence of S_4_^2−^. Furthermore, after the battery is discharged to 0.5 V, characteristic peaks appeared in the Raman spectra at 188 and 210 cm^−1^, indicating that Na_2_S is generated. The peak of 161.7 eV in the XPS spectrum also shows the presence of S^2−^.^6^ In addition, the peak at around 168 eV in Figure 5d–f corresponds to the S_2_O_3_^2−^.^36^, ^37^ The in situ Raman and ex‐situ XPS patterns indicate that during the discharge process, the sulfur mainly undergoes a two‐step conversion reaction process, the first step is converted into a long‐chain polysulfides (Na_2_S*_x_*, *x* = 4–8), which is then converted into short‐chain polysulfides (Na_2_S_2_/Na_2_S).

**Figure 6:**
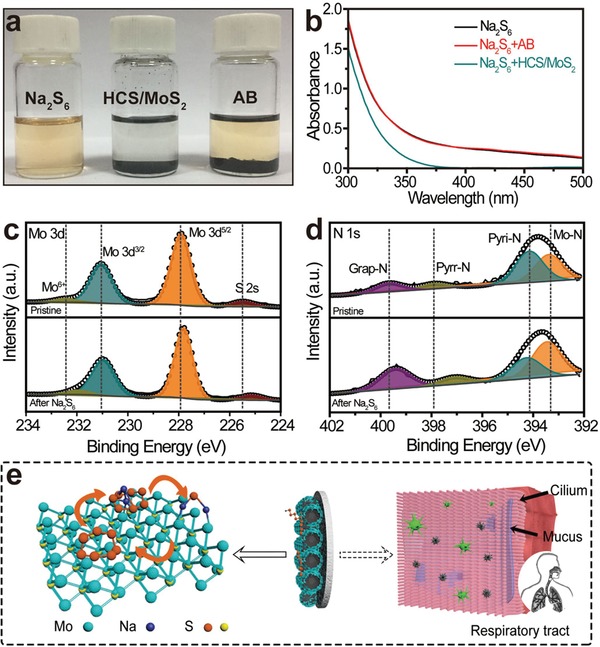
a) Polysulfides entrapment by the HCS/MoS_2_ and AB. b) UV‐Vis absorption spectra of Na_2_S_6_ solution before and after adding HCS/MoS_2_ or AB. The comparison of high‐resolution XPS spectra of c) Mo 3d and d) N 1s of the pure HCS/MoS_2_ and HCS/MoS_2_ + Na_2_S_6_ composite. e) Schematic diagram of the absorption and respiratory cilia.

The adsorption capacity of HCS/MoS_2_ is verified by the polysulfide adsorption test, ultraviolet‐visible absorption and XPS test. It can be seen from **Figure** **6**a that the initial color of the Na_2_S_6_ solution is light yellow, the color of the solution remains essentially unchanged after adding AB. The color of the solution changed from light yellow to colorless and transparent after adding HCS/MoS_2_. Figure 6b shows the change of Na_2_S_6_ concentration after adding AB and HCS/MoS_2_. The absorbance intensity of Na_2_S_6_ solution with HCS/MoS_2_ is much weaker than that of Na_2_S_6_ solution with AB, indicating that Na_2_S_6_ has a strong interaction with HCS/MoS_2_. In addition, the Na^+^ will intercalate into the layers of MoS_2_ between 0.5 and 2.8 V to form the sodiated MoS_2_ that can also adsorb the polar polysulfide (Figure S8a, Supporting Information), similar to MoS_2_.

Figure 6c,d shows the changes of Mo 3d and N 1s valence state of the HCS/MoS_2_ composite before and after immersion in the Na_2_S_6_ solution. The binding energies at 225.5, 228, 231, and 232.5 eV in the Mo 3d spectrum correspond to S 2s, Mo 3d^5/2^, Mo 3d^3/2^, and Mo^6+^, respectively. The peaks of N 1s reveal peaks of pyridine‐N (394.2 eV), pyrrolic‐N (398 eV), and graphitic‐N (399.7 eV). The peak of N 1s at the binding energy of 393.4 eV can be attributed to the Mo‐N coupling phase.^38^ After the HCS/MoS_2_ composite is contacted with Na_2_S_6_, since electrons are transferred from Na_2_S_6_ to Mo and N atoms, the Mo 3d and N 1s spectra are shifted to lower binding energy, indicating that there is a strong chemical interaction between HCS/MoS_2_ and the Na_2_S_6_. Figure 6e shows the adsorption of metal atoms with sodium polysulfides on polar MoS_2_ nanosheets. Interestingly, the effect of HCS/MoS_2_ modification is similar to that of cilia widely distributed in human respiratory tract. Along with the human respiration, pollutants such as dust and bacteria in the air can easily enter the respiratory tract. At this time, cilia are distributed on the surface of the respiratory tract, secreting mucus and preventing pollutants from entering the blood and cells. This process is similar to that of the HCS/MoS_2_ composite absorption of NaPSs by the modified glass fiber.

## 3 Conclusion

In summary, the composite of hollow carbon spheres coated with polar MoS_2_ has been designed and synthesized. On the one hand, the synthesized HCS/MoS_2_ composite as the S host can not only improve the conductivity of S, but also alleviate the volume expansion in the charge/discharge process. Carbon spheres and MoS_2_ provide the first defense against polysulfides spill. On the other hand, the HCS/MoS_2_ coated on the surface of the glass fiber can prevent the shuttle of polysulfides, similar to the cilia in the respiratory tract to block pollutants. As a result, this double defense system significantly improves electronic conductivity and blocks polysulfides shuttle, thereby improving rate performance and cycle stability of room‐temperature Na–S batteries.

## Conflict of Interest

The authors declare no conflict of interest.

## Supporting information

Supplementary
