# Supplementary material for: Design and Construction of Sodium Polysulfides Defense System for Room‐Temperature Na–S Battery
Source: Adv Sci (Weinh). 2019 Sep 30;6(23):1901557. doi: 10.1002/advs.201901557 (PMC6891912; doi:10.1002/advs.201901557)
Supplement: Supplementary file 1 — Supplementary [file ADVS-6-1901557-s001.pdf]

Copyright WILEY-VCH Verlag GmbH & Co. KGaA, 69469 Weinheim, Germany, 2019.

ADVANCED SCIENCE
Open Access

Supporting Information

for *Adv. Sci.,* DOI: 10.1002/advs.201901557

Design and Construction of Sodium Polysulfides Defense System for Room-Temperature Na–S Battery

Tingting Yang, Bingshu Guo, Wenyan Du, Muhammad Kashif Aslam, Mengli Tao, Wei Zhong, Yuming Chen, Shu-Juan Bao, Xuan Zhang,* and Maowen Xu**

Copyright WILEY-VCH Verlag GmbH & Co. KGaA, 69469 Weinheim, Germany, 2019.

Supporting Information

Design and construction of sodium polysulfides defense system for room-temperature Na-S battery

Tingting Yang, Bingshu Guo, Wenyan Du, Muhammad Kashif Aslam, Mengli Tao, Wei Zhong, Yuming Chen, Shu-Juan Bao, Xuan zhang*, and Maowen Xu**

Experimental Section

Preparation of SiO_2: 5 mL of cetyltrimethylammonium bromide (CTAB) solution (20 mg mL^{-1}) and 6.4 mL of NaOH solution (6 mg mL^{-1}) solution were added to the mixture solution of H_2O and N,N-dimethylformamide (DMF) (in a volume ratio of 1:1), respectively. The solution was then sonicated for 5 min at room temperature. After the methanol solution of tetraethyl orthosilicate (TEOs) (1.2 mL TEOs was dissolved in 4 mL methanol) was added to the above solution. After stirring for 6 h, it was washed with deionized water and ethanol several times. The SiO_2 powder was collected by centrifugation and dried overnight in an oven at 60 ℃.

Preparation of HCS: 60 mg of SiO_2 nanospheres were ultrasonically dispersed in 400 mL of Tris-buffer solution (10 mM, pH=8.5), and 180 mg of dopamine hydrochloride (DAH) was added after dispersion. After stirring for 12 h, the SiO_2@PDA nanospheres were collected by centrifuging and washing with deionized water and ethanol. The obtained SiO_2@PDA nanospheres were dried in an oven at 60 ℃ and then pyrolyzed at 700 ℃ for 2h in H_2/Ar (10:90 v/v) flow. Finally, the pyrolyzed sample was stirred at 80 ℃ for 3 h in HF (5 wt%) solution, washed with deionized water and ethanol, and dried at 60 ℃ for 12 h and finally HCS was obtained.

Preparation of HCS/MoS_2: In a typical procedure, HCS (10 mg) was sonicated in N,N-dimethylformamide (DMF) (30 mL) until a homogeneous black solution was formed. Tetrathiomolybdate ammonium (10 mg) was added to the solution and dispersed for 0.5 h.

The solution was then transferred to a 50 mL Teflon-lined stainless steel autoclave, sealed and heated at 210 ℃ for 15 h. After cooling to room temperature, the resulting product was extensively washed by water and ethanol and dried overnight at 60 ℃ in a vacuum oven, and then pyrolyzed at 700 ℃ for 2 h in a H_2/Ar (10:90 v/v) flow to obtain the composite with high crystallinity and purity of MoS_2.

Preparation of S@HCS/MoS_2: HCS/MoS_2 and S were mixed and ground in the weight ratio of 1:1, and then the mixture was annealed at 155 ℃ for 12 h in a tube furnace at a certain heating rate. After cooling down, the final S@HCS/MoS_2 composite was obtained.

Preparation of Na_2S_6 solution: The Na_2S and S were weighed in the mass ratios of 1:5, respectively and added to 20 mL of tetraethylene glycol dimethyl ether (TEGDME) solvent. The Na_2S_6 (0.067M) was obtained by stirring the above solution at 80 ℃ for overnight. The solution needs to be further dilution before the adsorption experiment.

Material Characterization: The morphology and structure were observed by field emission scanning electron microscopy (FESEM, JSM-7800F, Japan), transmission electron microscopy (TEM, JEM-2100, Japan) and scanning electron microscopy (SEM, JSM-6510LV). The composition of the products was analyzed by energy dispersive spectroscopy (EDS, JEOL-6300F). The thickness of the MoS_2 nanosheets was determined by an atomic force microscope (AFM, Bruker-Icon SPM). Powder XRD patterns were recorded using X-ray Diffraction (XRD, MAXima-X XRD-7000) in the range of 10°-80° degree 2θ with the rate of 5° min^{-1}. The pore structure of the composites were tested by Brunauer-Emmett-Teller method (BET, Quantachrome Instruments, USA). The TGA test was performed using an SDTQ600 analyzer (TA instrument) in the nitrogen atmosphere at 600 ℃ with a heating rate of 10 ℃ min^{-1}. The UV-visible spectrum was measured using a Shimadzu UV-2550 spectrophotometer. X-ray photoelectron spectroscopy (XPS) was performed using a Thermo Scientific ESCALAB 250Xi electron spectrometer.

In-situ measurements: The in-situ Raman cell was bought from EL-CELL Germany. The in-situ Raman was collected with Lab-RAM HR Evolution (Horiba) Raman microscope, with excitation 532 nm laser wavelengths. The spectra were collected when the in-situ Raman cell was discharged/charged at a sweep rate of 1 mV s^{-1} using a computer controller (CHI 660D).

Electrochemical Characterization: The electrode was fabricated by slurry coating process. The working electrodes were prepared by coating the slurry ,which was made by mixing of 80% of S@HCS/MoS_2, 10% of acetylene black (AB), and 10% of polyvinylidene difluoride(PVDF) in N-methyl-2-pyrrolidone (NMP) and pasted on the aluminum foil and then dried in vacuum at 60 ℃ overnight. The loading of S is about 0.5-1 mg cm^{-2}. CR 2032-coin cells were assembled in an argon-filled glove box using sodium metal as the counter electrode and the separators were HCS/MoS_2 coated glass fiber membrane (Whatman GF/A), which was obtained by coating the slurry of 80% of HCS/MoS_2 and 20% of PVDF on glass fiber (Whatman GF/A) followed by drying at 120 ℃ under vacuum for 12 h. The coating thickness of the HCS/MoS_2 is about 10 μm. The electrolyte used was 1 M $NaClO_4$ dissolved in tetraethylene glycol dimethyl ether (TEGDME). The cells were cycled at various current densities between 0.5 and 2.8V on a Land cycler (Wuhan Kingnuo Electronic Co., China). Cyclic voltammetry (CV) was performed at a scan rate of 0.1 mV s^{-1} using a CHI 660c electrochemical workstation (Shanghai Chenhua, China). The values of all specific capacities were calculated from the mass of active substance S. Electrochemical impedance spectroscopy (EIS) test was performed using a Zahner electrochemical workstation.

Supplementary Figures

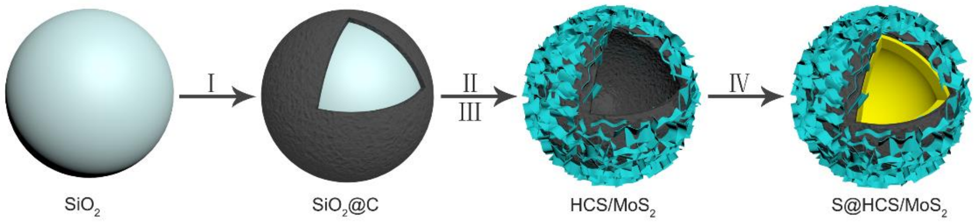

Figure S1. Illustration of the synthesis of the S@HCS/MoS_2 composite.

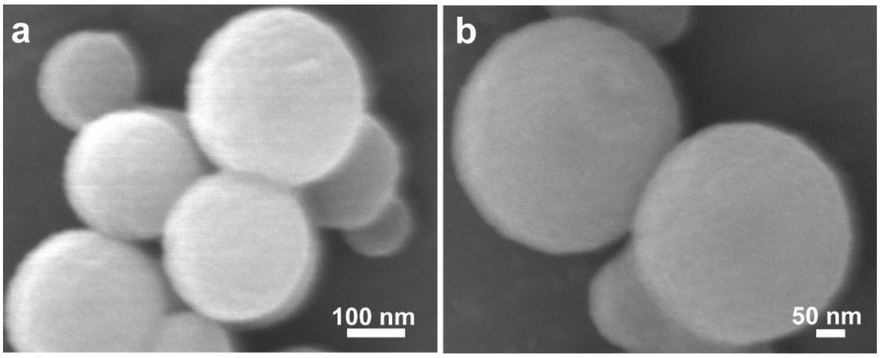

Figure S2. FESEM images of SiO_2 spheres.

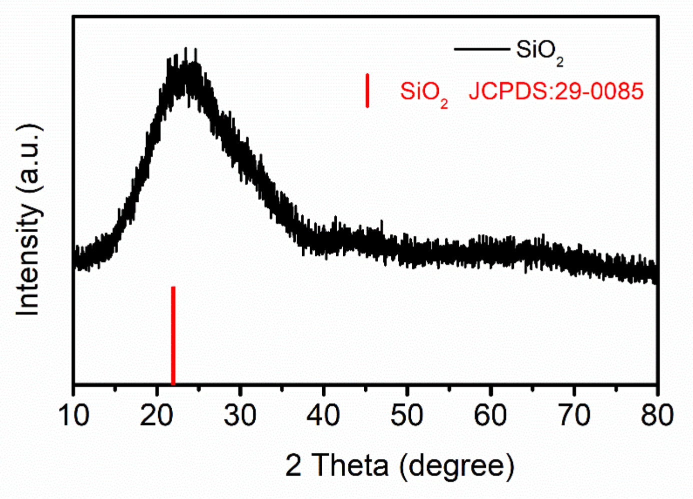

Figure S3. XRD spectrum of SiO_2 spheres.

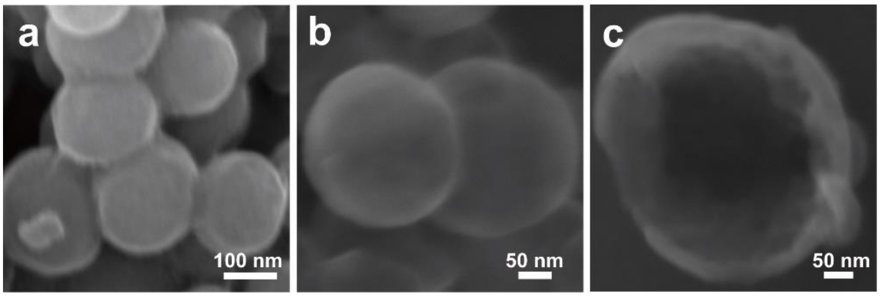

Figure S4. FESEM images of HCS.

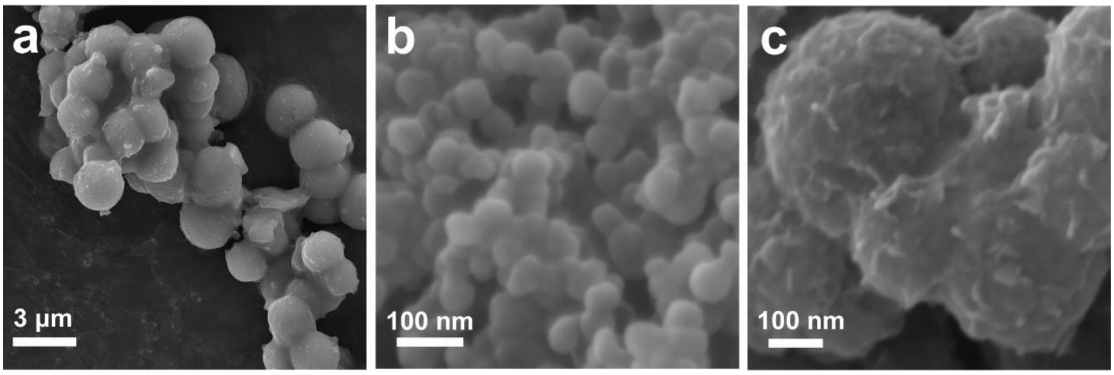

Figure S5. FESEM images of SiO_2 using (a) DMF solvent and (b) H_2O solvent and (c) HCS/MoS_2

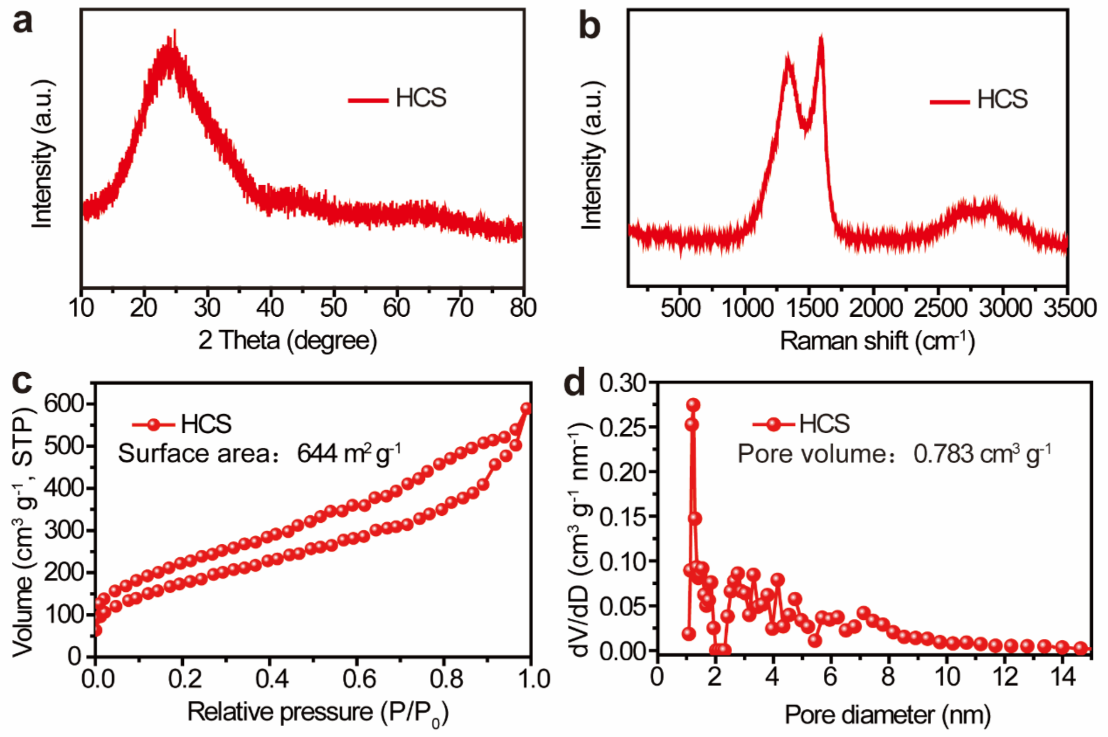

Figure S6. (a) XRD, (b) Raman spectrum, (c) BET adsorption and desorption curves, and (d) aperture distribution curve of HCS composite.

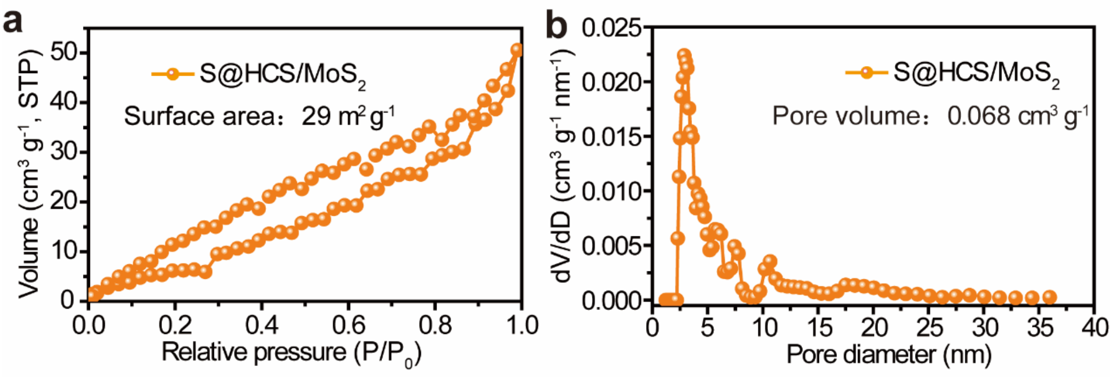

Figure S7. (a) BET adsorption and desorption curves and (b) aperture distribution curve of the HCS/MoS_2 composite.

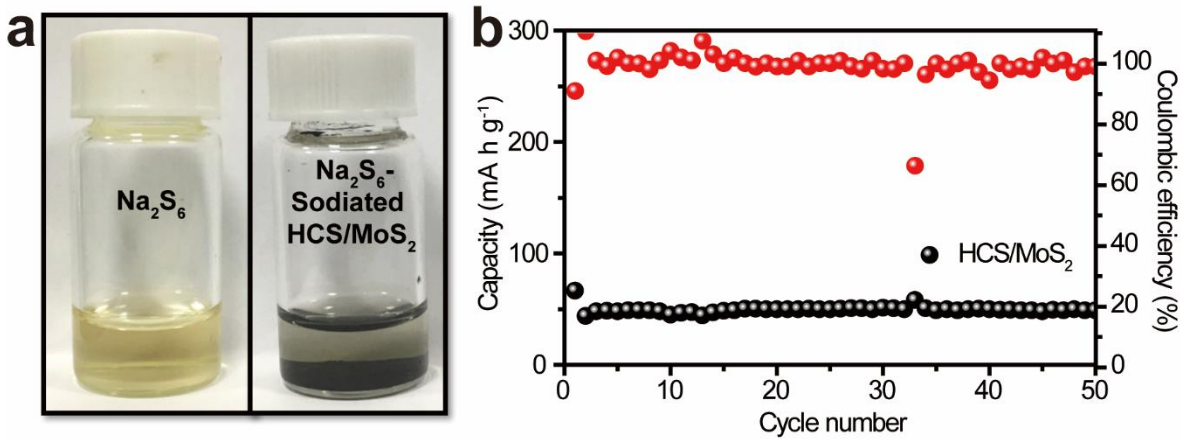

Figure S8. (a) Polysulfides entrapment by the sodiated HCS/MoS_2 and (b) Cycling performance of the HCS/MoS_2 composite electrode.

In view of the role of MoS_2 in the battery, HCS/MoS_2 was used to assemble the battery to discharge to 0.5V. Then, the battery was disassembled and the sodiated HCS/MoS_2 electrode was placed in Na_2S_6 solution after washing with tetraethylene glycol dimethyl ether (TEGDME) solvent. After standing for a period of time, the color of Na_2S_6 solution changes from pale yellow to colorless (Figure S8a), indicating that the sodiated MoS_2 (Na_xMoS_2, $x<2$) can also adsorb the polar polysulfide, similar to MoS_2.

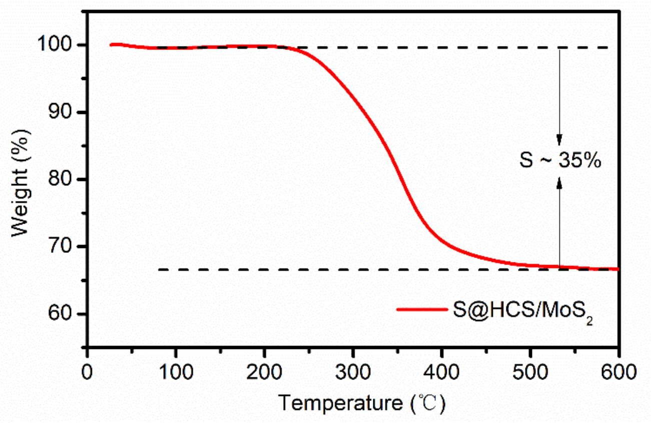

Figure S9. TGA curve of the S@HCS/MoS_2 with common glass fiber.

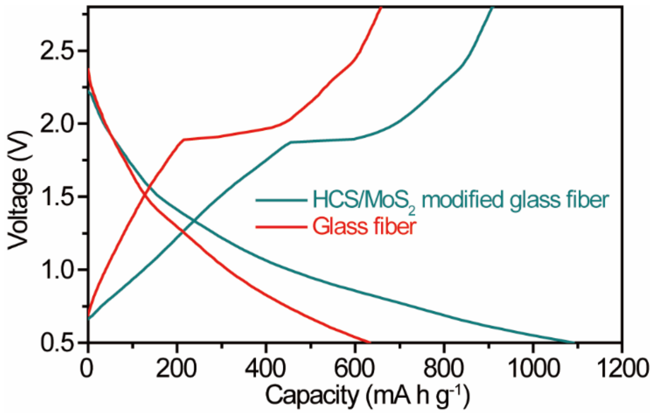

Figure S10. Voltage profiles of the S@HCS/MoS_2 composite electrode with modified glass fiber and glass fiber.

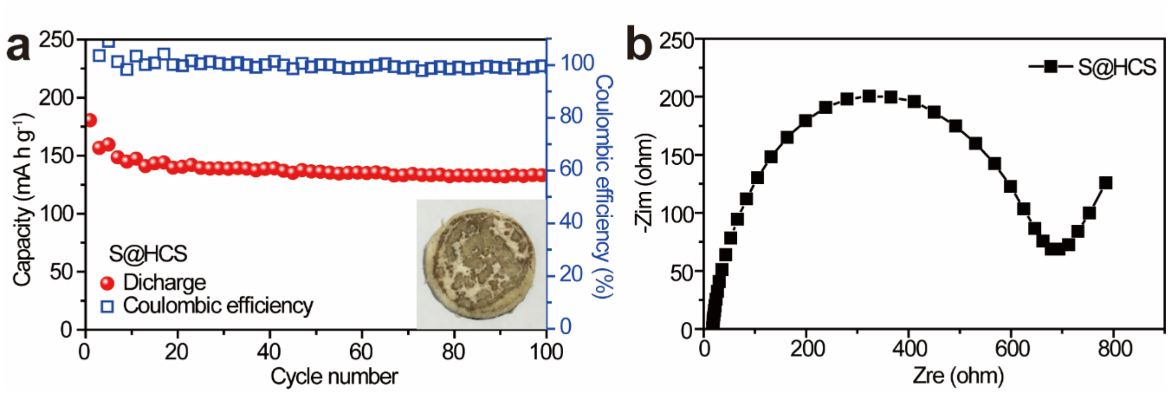

Figure S11. (a) Cycling performance of the S@HCS composite electrode, the inset is a photo of the corresponding separator after cycles. (b) Nyquist plots of the S@HCS electrode before cycle.

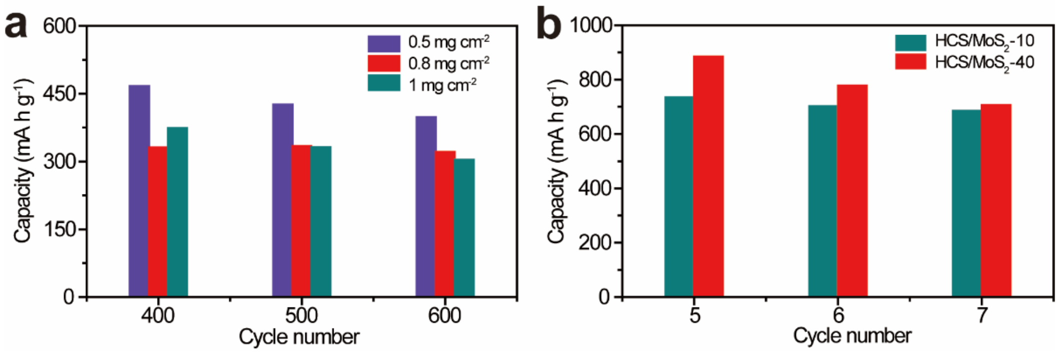

Figure S12. Cycling performance of the battery with increase of the thickness of the HCS/MoS_2 layers in (a) cathode and (b) separator.

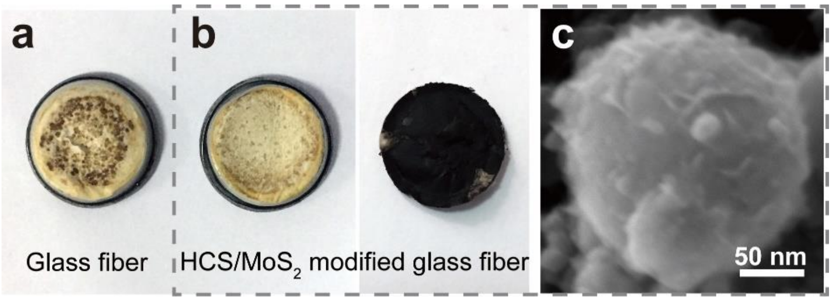

Figure S13. (a) The unmodified glass fiber after cycles, (b) the HCS/MoS_2 modified glass fiber after cycles, and (c) FESEM image of the S@HCS/MoS_2 composite after cycles.

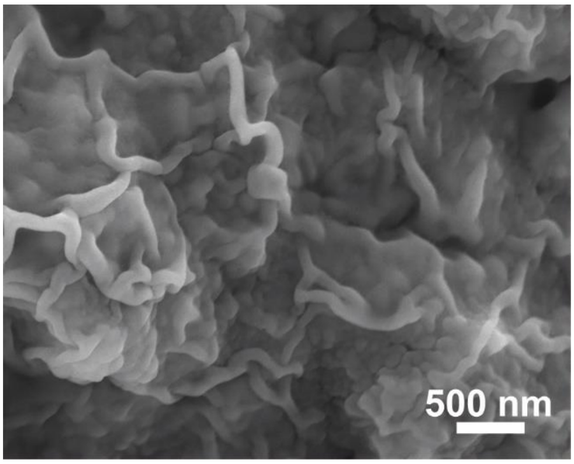

Figure S14. FESEM image of HCS/MoS_2 modified separator after cycles.

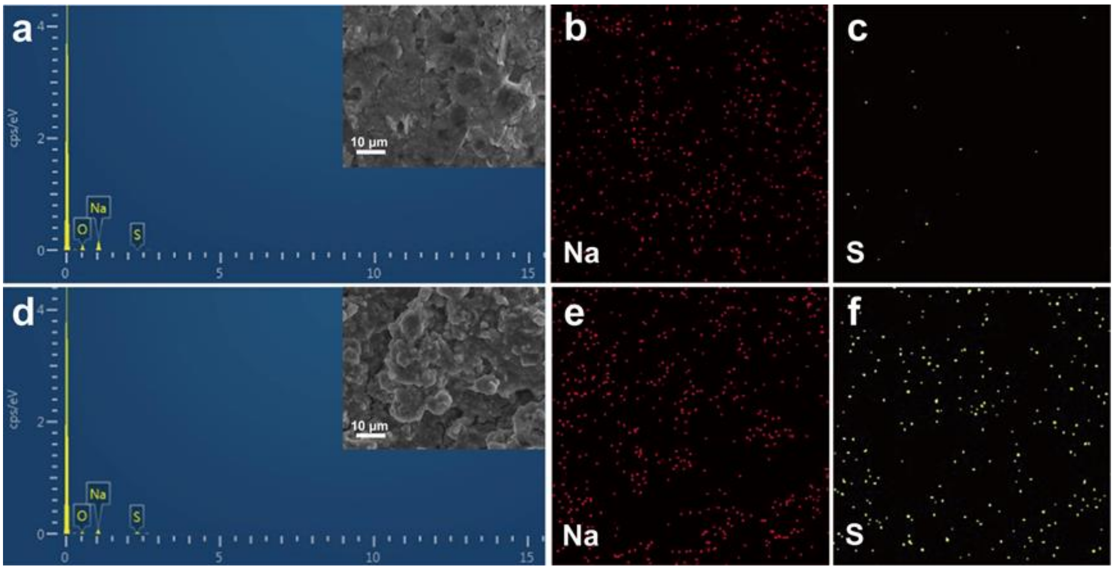

Figure S15. EDS elemental mappings and EDS spectra of Na from (a-c) the S@HCS/MoS_2 electrode with coating after cycles and (d-f) glass fiber electrode after cycles, insets are SEM images of the corresponding Na foil.

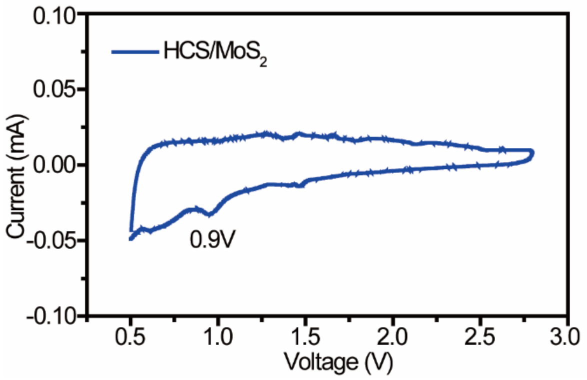

Figure S16. CV curve of the HCS/MoS_2 composite.

Table S1. The systematic comparison of the performance between our work and previously reported materials.

Materials	Electrolyte	S	Rate	Cycle performance Final capacity (mA h g^{-1}) /cycle number
S@HCS/MoS_2 with modified glass fiber	1 M $NaClO_4$ in TEGDME	44wt%	1 C	246 / 1000
hollow nano-Na_2S[1]	1 M $NaPF_6$ in Diglyme: DOL (1:1 by volume) with 0.08 M Na_2S and P_2S_5	68wt% (Na_2S)	1.4 A g^{-1}<br>2.1 A g^{-1}	600 / 100<br>400 / 100
Sulfur infiltrated spheres (S@C)[2]	$NaPF_6$ salts in TEGDME	35wt%	1 C	300 / 1500
S Nanosheets @Cu foam-150[3]	1 M $NaClO_4$ in PC: EC (1: 1 by volume) with 5wt% FEC	29.5wt%	50 mA g^{-1}	377 / 5
HCS-S[4]	$NaCF_3SO_3$ (molar ratio of 4 : 1) in TEGDME	56wt%	0.1 C	550 / 20
CNT@GNR/S[5]	1 M $NaClO_4$ PC: EC (1:1 by volume)	63wt%	0.2 C	350 / 300
HSMC-Cu-S[6]	1 M $NaClO_4$ in EC: DMC (1:1 by volume)	50wt%	0.03C	610 / 110
CFC/S-2[7]	1.5 M $NaClO_4$ and 0.2 M $NaNO_3$ in TEGDME	24.4wt%	0.1C	120 / 300
S@iMCHS[8]	1 M $NaClO_4$ in PC: EC (1: 1 by volume) with 5wt% FEC	46wt%	100 mA g^{-1}	292 / 200

[1] C. L. Wang, H. Wang, X. F. Hu, E. Matios, J. M. Luo, Y. W. Zhang, X. Lu, W. Y. Li, *Adv. Energy Mater.* **2018**, 1803251.

[2] R. Carter, L. Oakes, A. Douglas, N. Muralidharan, A. P. Cohn, C. L. Pint, *Nano Lett*. **2017**, *17*, 1863.

[3] B. W. Zhang, Y. D. Liu, Y. X. Wang, L. Zhang, M. Z. Chen, W. H. Lai, S. L. Chou, H. K. Liu, S. X. Dou, *ACS Appl. Mater. Interfaces.* **2017**, *9*, 24446.

[4] D. J. Lee, J. W. Park, I. Hasa, Y. K. Sun, B. Scrosatia, J. Hassoun, *J. Mater. Chem. A.* **2013**, *1*, 5256.

[5] A. P. V. K. Saroja, K. Muthusamy, R. Sundara, *Adv. Mater. Interfaces.* **2019**, 1801873.

[6] S. Y. Zheng, P. Han, Z. Han, P. Li, H. J. Zhang, J. H. Yang, *Adv. Energy Mater.* **2014**, 1400226.

[7] Q. Q. Lu, X. Y. Wang, J. Cao, C. Chen, K. Chen, Z. F. Zhao, Z. Q. Niu, J. Chen, *Energy Storage Materials*. **2017**, *8*, 77.

[8] Y. X. Wang, J. P. Yang, W. H. Lai, S. L. Chou, Q. F. Gu, H. K. Liu, D. Y. Zhao, S. X. Dou, *J. Am. Chem. Soc.* **2016**, *138*, 16576.
